# Evaluation of single-cell genomics to address evolutionary questions using three SAGs of the choanoflagellate *Monosiga brevicollis*

**DOI:** 10.1038/s41598-017-11466-9

**Published:** 2017-09-08

**Authors:** David López-Escardó, Xavier Grau-Bové, Amy Guillaumet-Adkins, Marta Gut, Michael E. Sieracki, Iñaki Ruiz-Trillo

**Affiliations:** 1 Institut de Biologia Evolutiva (CSIC-Universitat Pompeu Fabra), Passeig Marítim de la Barceloneta 37-49, 08003 Barcelona, Catalonia Spain; 2 Departament de Genètica, Microbiologia i Estadística, Universitat de Barcelona, Barcelona, Catalonia Spain; 3 CNAG-CRG, Centre for Genomic Regulation (CRG), Barcelona Institute of Science and Technology (BIST), Barcelona, Spain; 4 Universitat Pompeu Fabra (UPF), Barcelona, Spain; 5 National Science Foundation, Arlington, VA USA; 6 ICREA, Pg. Lluís Companys 23, 08010 Barcelona, Spain

## Abstract

Single-cell genomics (SCG) appeared as a powerful technique to get genomic information from uncultured organisms. However, SCG techniques suffer from biases at the whole genome amplification step that can lead to extremely variable numbers of genome recovery (5–100%). Thus, it is unclear how useful can SCG be to address evolutionary questions on uncultured microbial eukaryotes. To provide some insights into this, we here analysed 3 single-cell amplified genomes (SAGs) of the choanoflagellate *Monosiga brevicollis*, whose genome is known. Our results show that each SAG has a different, independent bias, yielding different levels of genome recovery for each cell (6–36%). Genes often appear fragmented and are split into more genes during annotation. Thus, analyses of gene gain and losses, gene architectures, synteny and other genomic features can not be addressed with a single SAG. However, the recovery of phylogenetically-informative protein domains can be up to 55%. This means SAG data can be used to perform accurate phylogenomic analyses. Finally, we also confirm that the co-assembly of several SAGs improves the general genomic recovery. Overall, our data show that, besides important current limitations, SAGs can still provide interesting and novel insights from poorly-known, uncultured organisms.

## Introduction

In the last decade, molecular techniques based in the sequence of 18 S rDNA gene have deciphered an impressive amount of hidden eukaryotic diversity^1–5^. However, most of the genomic information available from eukaryotes is still biased towards a handful of culturable organisms that do not fully cover the biological diversity described by molecular studies^6^. The genomes of these uncultured eukaryotic lineages can contain key evolutionary information both to infer a more complete eukaryotic tree of life (ETOL)^7^ and to better reconstruct the evolution from the Last Eukaryotic Common Ancestor (LECA)^8^ to the extant species.

Single-cell genomics (SCG) was initially proposed as a very promising technique to get the genomes of uncultured taxa directly from the environment^9,10^. In contrast to metagenomics data, SCG allows to recover genomic DNA from one single cell. Single cells from the environment can be isolated using different techniques such as micromanipulation^11^, microfluidics^12^ and by using a Fluorescence Activated Cell Sorting (FACS)^10^. Cell isolation is then followed by cell lysis and a whole genome amplification (WGA) step^10^. The discovery of chemolithoautotrophy pathways in uncultured Proteobacteria^13^, or the proposition of two new prokaryotic superphyla^14^, are two examples of promising findings obtained thanks to single-cell genomics in prokaryotes.

However, single-cell genomics have also some important drawbacks that challenge their use in all microbial forms, including eukaryotes. For example, the sample can suffer an amplification bias at the WGA, as well as the appearance of artefacts or genome loss^15,16^. Multiple Displacement Amplification (MDA)^17–19^, which uses a high fidelity phi29-polymerase^20^, is the standard method used for microbial applications^21^. Even though MDA leads to lower amplification error rates and lower contaminations compared to other methods^15,16^, it also provides uneven WGA amplifications that can be even higher than with other WGA methods^22^. The MDA method provides between 5–100% of genome completeness in bacteria, with an average of around 40%^14^. Fewer studies have been done in unicellular eukaryotes. A recent work done on the parasite *Cryptosporidium* recovered most of the genome^23^. However, *Cryptosporidium* can be purified from fecal samples and has a rather small genome compared to most eukaryotes. Few studies have so far used single-cell amplified genomes from eukaryotic samples directly obtained from the enviroment^24–28^. The genome recovery on those studies varies widely (between 9–55%). Interestingly, a study focused in an uncultured group of marine stramenopiles (MAST)^29^, showed that by co-assembling different SAGs from different cells the genome recovered increased substantially^28^. Thus, it remains yet unclear the full potential of this methodology and how to best approach the analyses of the data recovered from SAGs. These are important questions because the scientific community is generating more SCG data. A good example is that more than 500 SAGs belonging to uncultured eukaryotic lineages^6^ have been already generated from the TARA oceans expedition^30^. These SAGs could potentially provide novel insights into eukaryotic evolution, but we need to understand what can we do with the data generated as well as be aware of the best potential strategies for genome assembly and genome annotation.

To provide insights into the potential of SAGs, we here analyzed three different SAGs obtained from uncultured samples, but corresponding to one single species, whose genome is of an average protist size and already sequenced. In particular, we analyzed three SAGs from the TARA oceans expedition that belong to the choanoflagellate *Monosiga brevicollis*, whose genome is already sequenced and annotated (strain MX1, ATCC PRA-258^31^). The average size of most of the published genomes from unicellular eukaryotes is 61.1 Mb (±9.76 Mb)^32^, including the diminutive microsporidians (2.5 Mb in *Encephalitozoon cuniculi*)^33^ and the larger genome of the oomycete *Phytophthora infestans* (228.5 Mb)^34^. Genome lengths of a few taxa can be even higher, as the ones reported for the foraminiferan *Reticulomyxa filosa* (320 Mb)^35^ or the amoebozoan *Amoeba dubia* (estimated at 670,000 Mb)^33^. In any case, the genome of *M*. *brevicollis* is 41.6 Mb, which makes it an ideal candidate for our purposes. Thus, we tested different conditions of *de novo* genome assembly and genome annotation, and checked the percentage of gene and proteins domains recovery. Our data demonstrates that, although there are important biases, some bioinformatic pipelines can adequately increase the genomic information recovered, being at least useful for phylogenomic analyses. We also show that co-assembly of several SAGs improves the general genomic recovery.

## Results

We analyzed three independent environmental SAGs (henceforth called MB1, MB2 and MB4), which had 99.6-100% 18S rRNA nucleotide identities with the 18S rRNA of the choanoflagellate *M*. *brevicollis*. SAGs were isolated from two different geographical locations; the Arabian sea (MB1 and MB4) and the Maldives bay (MB2) (Supplementary Table S1). We performed library preparation and sequenced them with Illumina MiSeq (see Methods). After a strict quality trimming, we ended up with a total of 24–34 million reads representing 117–163X of genome sequencing depth for each individual SAG (Supplementary Table S2). Furthermore, we also analysed the co-assembly of all reads coming from the three SAGs having been pooled together (henceforth “pooling”).

**Figure 1:**
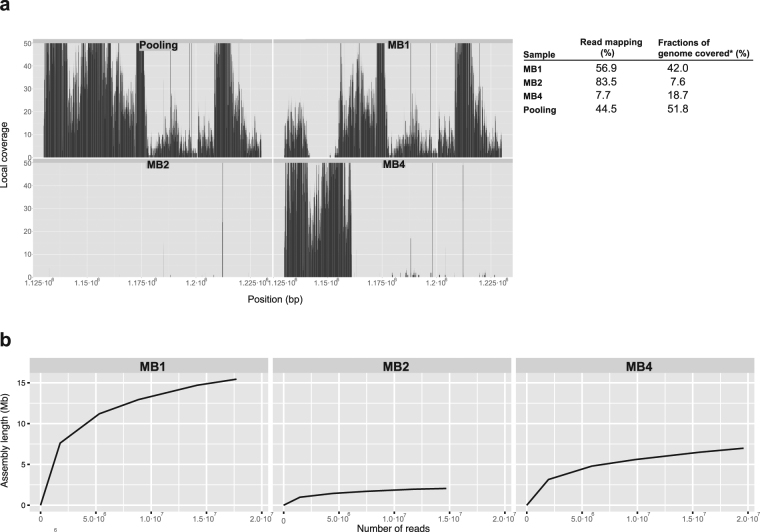
SAGs read mapping and downsampling analysis. (**a**) Read mapping of each SAG (MB1, MB2, and MB4), plus all reads pooled together (Pooling) on the *M*. *brevicollis* genome done with bowtie2 (see methods). The figure shows the read mapping a on a window of the reference genome, within the scaffold CH991545. This genomic window is representative of the overall distribution of the read mapping along the genome. X-axis indicates the position on the scaffold CH991545, and the Y-axis shows the depth of read mapping. The figure shows up to 50x of coverage, but note that there are positions with more than 1000x of read mapping. The table on the right shows the percentage of reads that map to the reference genome and the percentage of the genome length they cover. (**b**) Downsampling analyses for all three SAGs, showing the length of the assembly obtained on the Y-axis, and the number of reads used on X-axis. The reads used in each analysis were a subsampling of 10%, 30%, 50%, 80% and the total number reads of each SAG obtained after an strict quality trimming (see results and methods).

As MDA can lead to the generation of chimeric DNA fragments^36^ and the amplification of sample contaminants, we first mapped these reads to the *M*. *brevicollis* reference genome and observed that the number of aligned reads varied widely among the different SAGs. MB2 had the highest percentage of reads mapping to the reference genome (83.5%), followed by MB1 (56.9%) and MB4 (7.7%) (Fig. 1a). However, the reads were not equally distributed across the length of the genome: MB1 covered up to 42% of the reference genome (even though it had less reads mapping to the genome), followed by MB4 (18.7%) and MB2 (7.6%). Therefore, even though MB2 presented a higher percentage of read mapping, those reads were extremely biased towards a few genomic regions (Fig. 1a). Thus, none of the SAGs covered all the reference genome, observing important differences between the different SAGs.

### Genome assembly: downsampling test

In order to check how the uneven distribution of read mapping affects the final genome assembly, we assembled different amounts of reads using SPAdes assembler^36^, which is designed for single-cell genomics data. The SPAdes assembler avoids the interference of artifacts, while it can deal with uneven genome coverage. This was indeed our case, since we had an excess of sequencing depth, but unequally distributed along the genome (Fig. 1a). We examined whether the addition of more reads would provide information on the non-amplified or poorly-covered genomic regions, thus increasing the final length of genome assembly. To test that, we downsampled the original sequencing libraries by randomly selecting different fractions of the total number of reads (10%, 30%, 50%, 80% and 100%), and by performing independent genome assemblies to observe how the assemblies length changed with each fraction of reads. Our downsampling test (Fig. 1b) showed that it is possible to know how well the WGA reaction worked with low sequencing depths (10–20X). Moreover, we observed that our SAGs were not completely saturated with 100% of reads, meaning that including more reads could potentially improve the length of the assembly. Thus, to test that and to perform our final assemblies, we went back to the original raw reads and performed a more relaxed quality trimming in order to maximise the number of available reads, while having their overall accuracy within ranges tractable by the HammerBayes error correction algorithm^37^ (>99% over 6-nucleotide sliding windows; see Methods), implemented in SPAdes^36^ assembler. This approach yielded longer assemblies in the three SAGs, while providing similar genome contiguity statistics (N50, L75) and similar incidences of artifacts/contamination (Supplementary Table S2). We therefore used these assemblies in subsequent analyses.

### Identification of contaminant scaffolds

**Figure 2:**
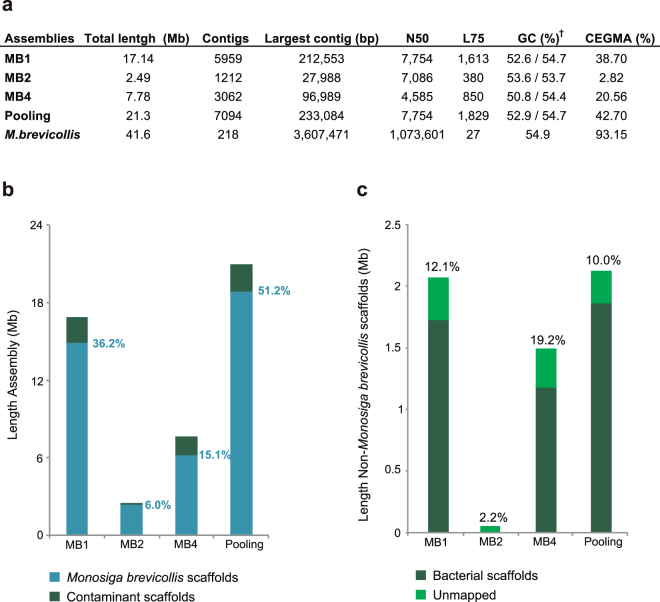
Assembly statistics. (**a**) Genome assembly statistics of the reference *M*. *brevicollis* genome, each individual SAGs and the pooled assembly; calculated using scaffolds longer than 500 bp. ^†^GC content is shown both for the complete assembly (left) and scaffolds that belong to *M*. *brevicollis* (right). (**b**) Assembly length of each SAG. Blue segments indicate the total length of scaffolds mapping to the reference *M*. *brevicollis* in each SAG and the pooled assembly (including the % of reference length covered by each SAG); dark green segments correspond to contaminant scaffolds. (**c**) Total length of the scaffolds that do not map to the reference *M*. *brevicollis* genome in each SAG and pooled assembly (including the % of contaminant scaffolds’ length in each assembly). Dark green indicates the scaffolds mapping to known bacterial sequences; light green indicates non-mapping scaffolds (BLASTn against Genbank; see Methods).

Once we had the final assemblies, we decided to investigate whether all scaffolds belonged to *M*. *brevicollis*, or whether there were some contaminant scaffolds. To this end, we aligned the assembled scaffolds to the reference genome. We found that most, but not all, of our scaffolds mapped to *M*. *brevicollis* genome (Fig. 2b). In particular, around 2–19% of each assembly did not belong to *M*. *brevicollis* reference genome (Fig. 2c).

Besides the possible appearance of contaminants during the WGA^15^, there is the fact that *M*. *brevicollis* is an heterotrophic organism that preys bacteria, so the collected cells could had engulfed bacteria. Indeed, a BLASTn search against NCBI non-redundant nucleotide database showed that these non-*Monosiga* scaffolds were mainly bacterial, but there were also some that did not map with any known sequence (Fig. 2c). Additionally, we performed a tetra-nucleotide frequency analysis to confirm our identification of contaminant scaffolds on the basis of nucleotide sequence composition (Supplementary Fig. S1). All the putative contaminant scaffolds clustered together among tetra-nucleotide frequency values (Supplementary Fig. S1). Moreover, as contaminant scaffolds were affecting the final G-C content values of the assembly (Supplementary Fig. S2); once they were identified and removed, we obtained the same G-C content (54%) as the reference genome of *M*. *brevicollis* ^31^ (Fig. 2a) (Supplementary Fig. S2). To further investigate the potential sources of contamination, we profiled the bacterial 16S rDNA genes present in our contaminant scaffolds by BLASTn similarity searches against Genbank. We found two OTUs shared between different SAGs, which correspond to uncultured soil or freshwater bacteria: the proteobacteria *Polynucleobacter* (present in MB1 and MB2) and one unclassified uncultured bacteria (present in MB1 and MB4). Furthermore, we identified six OTUs appearing only in one SAG: two without clear taxonomic affiliation (in MB2), two Alphaproteobacteria (one uncultured and the other belonging to *Methylobacterium* genus, found respectively in MB1 and MB2), one Deltaproteobacteria (a Desulfurimonadales from MB1) and one Bacteroidetes (MB1) (Supplementary Table S3). This opens the possibility that these bacteria came from contaminants during the WGA step or, alternatively, from putative bacteria engulfed or attached to *M*. *brevicollis* cells.

### SAGs assemblies compared to the reference genome

**Figure 3:**
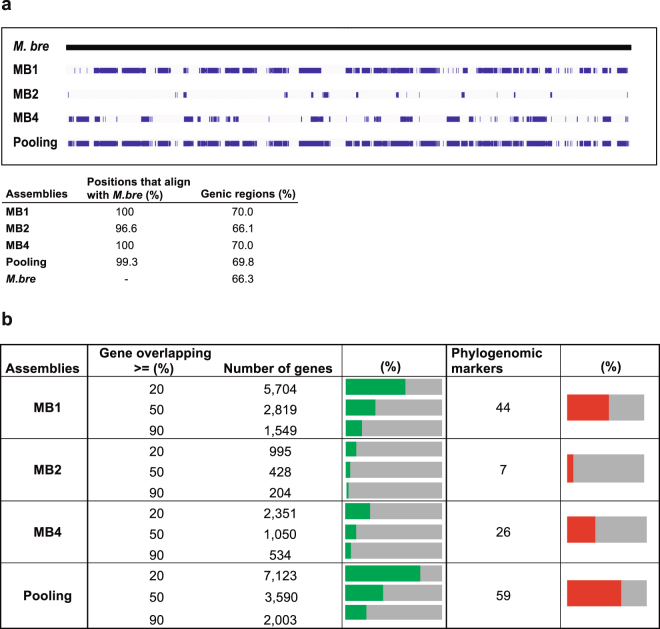
Scaffold mapping to reference genome and gene recovery. (**a**) Scaffold mapping of each SAG assembly and the pooling over the reference genome calculated with LAST, only the scaffolds bigger than 500 pb were used for analysis. It is shown the chromosome CH991544 of *M*. *brevicollis*’s genome as a representative of the whole scaffold mapping to the reference genome. The table summarizes the percentage of the alignment length compared with the size of the assembly of each SAG (*M*. *bre* for *M*. *brevicollis*) and the percentage that belongs to genic regions. (**b**) Number of genes recovered in each assembly comparing the scaffold mapping of the SAGs and the gene annotation of the reference genome. Different numbers of genes depending on the sequence completeness recovered (at least 90%, 50% or 20%) are shown. Green bar charts indicate the percentage compared to the total number of genes of the reference genome. On the right, the number of phylogenomic markers from Torruella *et al*.^38^ found in each assembly and the percentage they represent of the total phylogenomic dataset (red bar charts).

We aimed to compare the quality of our SAGs and pooling assemblies with the reference *M*. *brevicollis* genome (MX1 strain), in terms of completeness, contiguity and gene recovery. We used for that the alignment information between the reference genome and our final assemblies, which practically fully mapped with the reference genome (96.6–100%) (Fig. 3a). The genome completeness of our final assemblies was low (Fig. 2b). MB1 recovered 36.2% of the *M*. *brevicollis* genome (the highest individual SAG), while MB4 recovered 15.1% of the genome and MB2 only 6%. Interestingly, the co-assembly of all three SAGs presented higher genome completeness (46.1%) than MB1 alone (Fig. 2a). This is due to the fact that each individual SAG is the result of an amplification of different genomic regions (Fig. 3a). Moreover, the positions aligned to the reference genome by the three individual SAGs in total are practically the same than the positions recovered by the pooling assembly (99.1% of the positions recovered by the combination of the three individual SAGs are shared with the pooling assembly). However, pooling co-assembly allows the recovery of slightly more positions of the reference genome than the three individual SAGs merged together (accounting for 536 kb, a 2.9% of the pooling assembly’s length).

Next, we analyzed the rate of recovery of genic regions in our SAGs, compared with the reference *M*. *brevicollis* genome. First, we found that the percentage of coding regions in our assemblies was ~66–70%, similar to the percentage in the *M*. *brevicollis* genome (66%) (Fig. 3a). This suggests there is no a significant bias towards coding or non-coding regions in the amplification step. Then, we screened which percentage of *M*. *brevicollis* genic regions are present in our SAGs scaffolds. We plotted 3 different situations: 1) coding regions that are almost fully recovered (i.e. >90% complete), 2) coding regions that are at least half complete (>50% complete), and 3) highly fragmented coding regions (>20% complete). As expected, the pooling and MB1 assemblies have the highest numbers of recovered genes. However, these genes are highly fragmented: out of the 9,172 genes in *M*. *brevicollis* genome, MB1 assembly has 1,549 almost complete genes (>90% of the coding region aligning to the reference), and 5,704 fragmented genes (>20%) (Fig. 3b).

To better understand how different are the individual SAGs between them and compared to the original *M*. *brevicollis* genome (MX1 strain^31^), we calculated their average nucleotide identity (ANI). We found that they are highly similar (~99% ANI; Supplementary Fig. S3). Thus, according to Mangot and co-workers^28^, our SAGs are similar enough to be pooled together. On the other hand, the comparisons of each SAG against the reference *M*. *brevicollis* genome yielded lower sequence identity values (~95% ANI; Supplementary Fig. S3). Thus, our SAGs probably correspond to a different strain than the MX1 used for the genome sequence^31^. To further check whether those differences may be affecting our results, we performed a synteny analyis between the reference *M*. *brevicollis* and the pooled assembly (representative of all individual SAGs) (Supplementary Fig. S4). We identified local inversions in 55 scaffolds of the pooled assembly (covering 800 kb out of 21.3 Mb assembled, or 41.6 Mb in the reference), which spanned 48 genes (representing 0.71% of the genes recovered in the pooling assembly with at least 20% of completeness, Fig. 3b): 12 with >90% of gene completeness, 24 between 90-50% of gene completeness and 12 between 50-20% of gene completeness. Therefore, these rearrangements events have a minimal presence in our SAGs, and the few genes affected are not specially fragmented compared with the other genes recovered.

### Assessing the utility of SAGs for phylogenomic analysis

**Figure 4:**
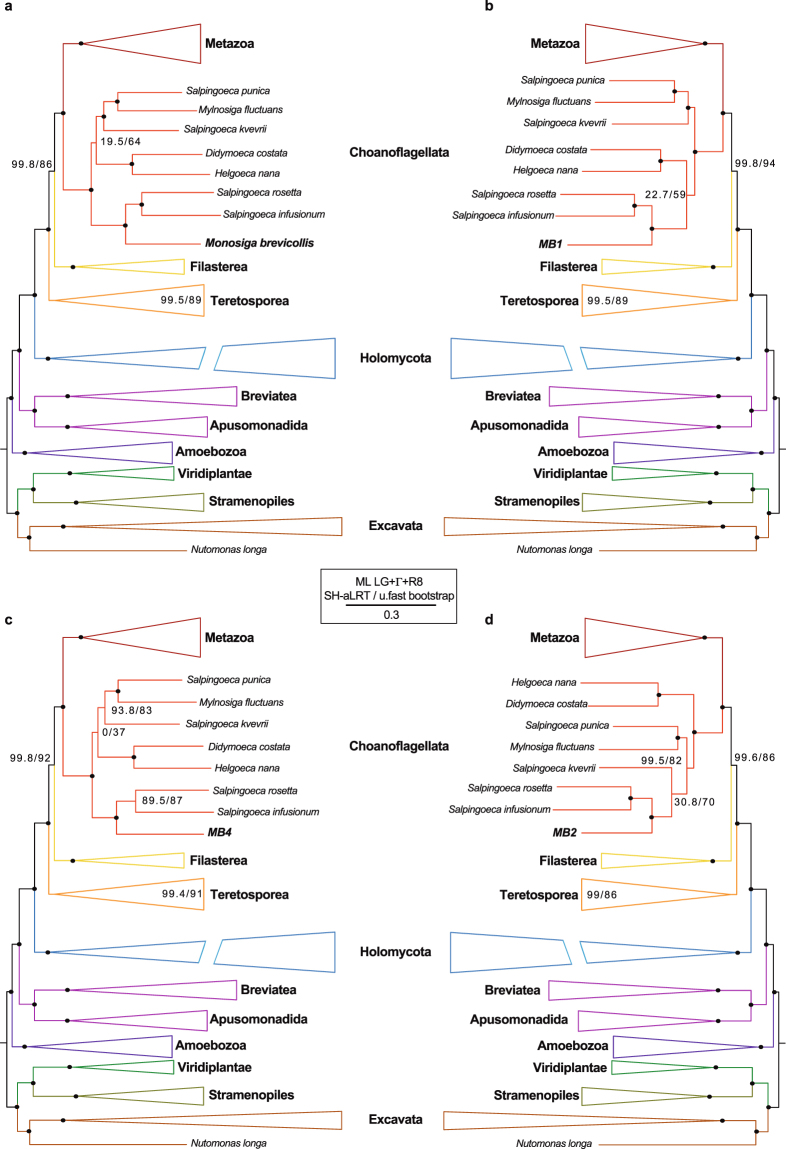
Phylogenetic placement of each SAGs. Phylogenetic trees based on 83-taxa matrix from our phylogenomic dataset^38^ (see Supplementary Figs 3, 4, 5 and 6) inferred by Maximum likelihood under the LG+ Γ free rate with 8 categories model. Split supports are bootstraps of single branch test (SH-aLRT, left number) and ultrafast bootstraps (right number) calculated with IQ-TREE. Split support values >90 of SH-aLRT bootstraps and >95% of ultra fast bootstrap computed with IQ-TREE are indicated by a bullet (•). Each tree A, B, C, D represents the phylogenetic position of the reference genome (**a**), the SAG MB1 (**b**), the SAG MB4 (**c**) and the SAG MB2 (**d**) respectively, calculated with the same parameters. Note that all SAGs and the reference genome fall in the same phylogenetic position, although there are some changes in the internal topology of choanoflagellates.

As we are specially interested in the use of SAGs for evolutionary studies, we checked whether enough phylogenetic markers were recovered to perform phylogenomic analyses. Specifically, we searched for the presence of protein domains of a phylogenomic matrix that had been used in a previous phylogenomic analysis of Opisthokonta^38^. Thus, we tested whether, in the hypothetical absence of a *M*. *brevicollis* reference genome, SAGs would contain enough gene markers to correctly place a putative new species in the eukaryotic tree of life. We found that MB1 and MB4 contain, respectively, 56% and 33% of the gene markers, and 8,655 and 4,601 of the ungapped positions within the phylogenomic alignment (out of 21,231 ungapped positions of the reference genome on the global dataset of 22,393 positions), which can be sufficient to perform a phylogenomic analysis. In contrast, the numbers of domains recovered in MB2 were very low (just 7 out of 78 protein domains comprising 1,689 ungapped positions compared to the 21,231 positions of the reference genome) (Fig. 3b). The phylogenetic analysis of each individual SAG correctly placed them within choanoflagellates, as sister-groups to *Salpingoeca rosetta* and *Salpingoeca infusionum* with high statistical support (Fig. 4b–d) (Supplementary Figs S5, S6 and S7), as it occurs with the reference genome (Fig. 4a) (Supplementary Fig. S8). Furthermore, a joint phylogenomic analysis of each SAGs and the reference *M*. *brevicollis* confirmed that all four genomes cluster together with maximal statistical support (and minimal internal amino-acidic differences (Supplementary Fig. S9)), thus confirming the consistency of each individual set of phylogenetic markers. It is worth mentioning, however, that the internal topology of choanoflagellates varies among each analysis, particularly in the less supported deeper nodes.

### Gene annotation in single-cell genomics

Once we had enough information from the assemblies, we tested different gene annotation methods in order to benchmark the possible outcomes depending on the *ab inito* gene predictors and strategies used. An important challenge to annotate single-cell amplified genomes, besides the fragmentation of the coding regions, is the lack of transcriptomic data to train the annotation algorithms. We here tried three different approaches to annotate the genomes: (1) *ab initio* annotation with Snap^39^; (2) annotation using Augustus^40^ trained with the complete proteins predicted with Snap (see Methods); and (3) annotation with Augustus trained with CEGMA (a set of 248 genes universally conserved in eukaryotes^29^) proteins, which is the method that have been used in other single-cell genomics studies^24,25,28^.

**Figure 5:**
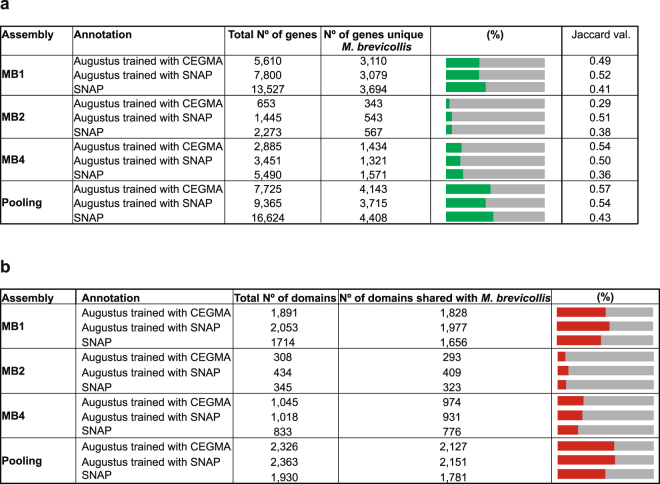
Gene annotations. (**a**) Summary of results from different gene annotation strategies: Augustus trained with CEGMA, Augustus trained with SNAP, and SNAP alone, carried out for each SAG, plus the pooling. The column *total number of genes*, are the numbers of genes obtained in each annotation. The column *number of genes unique M*. *brevicollis*, represents the number of different genes of *M*. *brevicollis* recovered in our SAG annotations, calculated using BLASTp between the annotated proteins of our SAGs and the proteins of the reference genome (see Methods). Green bar charts indicate the percentage of the number of unique genes of *M*. *brevicollis* found in the each annotation compared with the total number of genes of *M*. *brevicollis*. (**b**) Protein domain information obtained in each annotation. All the columns are analogous with the columns of A) but depicting the information for protein domains. The third column of shared domains was calculated by running Venn diagrams (see Methods). Red bar charts indicate the percentage of the protein domains shared between SAG annotations and the *M*. *brevicollis* reference genome compared with the total number of protein domains of *M*. *brevicollis* reference genome.

We measured the accuracy of each SAG annotation by comparing its relative overlap with the reference *M*. *brevicollis* annotation, using the *J* statistic from the Jaccard test^41^ (*J* values range from 0 to 1, representing no-overlap and perfect intersection, respectively). We found that Augustus (trained with either Snap *ab initio* predictions or CEGMA proteins) performed better than Snap, providing the more accurate annotations (Snap *J* = 0.36-0.43, Augustus *J* = 0.50–0.54; Fig. 5 and Supplementary Fig. S10). In the best annotated SAG, MB1, we recovered a third of the original *M*. *brevicollis* proteins (33–40%). We found, however, that *ab initio* Snap annotations overestimate the number of genes, in some cases providing more than two times the number of genes annotated with Augustus. A closer look at these genes, however, showed that most of them were false positives not present in the reference annotation (Fig. 5a).

Finally, we annotated the protein domains present in each SAGs gene predictions (using HMM -based searches and the Pfam database). Specifically, we found that the lower quality of Snap-based gene predictions is also reflected by their lower number of annotated protein domains when compared to the Augustus-based predictions (Fig. 5b), an independent measure of annotation accuracy. Finally, we found that, for each SAG, the vast majority of its annotated domains were shared between all three annotation methods and the reference *M*. *brevicollis* genome (e.g., for the Pooling, 77–93%; see Supplementary Fig. S11).

## Discussion

We have here sequenced three environmental SAGs belonging to the choanoflagellate *M*. *brevicollis*, for which a reference genome sequence obtained from a culture is already available^31^. The aim is to show further light into both the potentialities and the drawbacks of current single cell genomics technologies when dealing with environmental cells. In addition, we have compared individual SAGs assemblies versus a co-assembly of all three SAGs and have tried different *de novo* assembly and *de novo* annotation strategies. Finally, we have tested the potential of the data for phylogenomics and gene content analyses. Overall, we provide a global picture of the potential downstream analyses to be performed when dealing with environmental SAGs.

As already shown in other analyses^28^, we observe an intrinsic bias for each SAG, probably due to biases during the WGA amplification reaction. It is worth mentioning that cell lysis and DNA denaturation can as well affect the outcome of the WGA^15,16^. We found that the presence of unmapped reads against the reference genome of *M*. *brevicollis* varies a lot among different SAGs (between 16.5–93.3%). Some SAGs present a lot of unmapped reads (93.3%), as is the case of the MB4 sample. These unmapped reads can be the effect of chimeric assemblages produced during the WGA^36^ or contaminant reads. We mapped all the reads to our contaminant scaffolds and we found that most of the unmmaped reads are due to contamination, concretely 84% of MB4 reads map to the contaminant scaffolds. That means that, in some cases, the contaminants can importantly affect the amplification depth of the sample. However, SAGs with less contaminant reads and more read depth, as MB2 (10.64% of contaminant reads, 83% of read mapping to the reference genome), can also produce assemblies with extremely low genome completeness (6% compared with 15.1% of MB4), due to high uneven coverage. In our best case, the MB1 SAG, the genome completeness is 36.2%, being some genomic regions highly sequenced while others had not reads. Thus, there is an important variance on the data obtained from each single-cell amplification procedure.

Our analysis demonstrates that low sequencing depths are sufficient to assess the *a priori* quality of a SAG by randomly downsampling reads and analysing the subsequent assemblies’ lengths. Thus, a short assembly length and a saturated curve in downsampling analysis indicates that a SAGs is of low quality. In fact, various metrics have been constructed, following this principle, to quantitatively predict the complexity of single-cell genomics libraries at low sequencing depth in human cancer cells^42^. Moreover, downsampling tests can be used to decide whether to sequence deeper or not. Our results suggest that in the case of a SAG whose assembly length is not saturated, additional sequencing (or less strict trimming) helps to increase in a 5–10% the assembly’s length. We also show that in SAGs with extreme low genome recovery, as is the case of MB2, more sequence depth would be a waste of resources. In our case, using a less strict trimming (see Methods) did not affect the general genome statistics and the appearance of more artifacts or contamination, while producing longer assemblies (Supplementary Table S2). Thus, using less strict trimming of the data seems a good approach that should be taken into account in single-cell genomics studies.

Co-assembly of different SAGs has already been suggested to be an interesting way to increase the genomic information of different SAGs corresponding to the same taxa. In particular, Mangot and co-workers^28^ found that by pooling 14 SAGs of MAST-4A they recovered more than 74% of CEGMA proteins. In our case, by pooling our three SAGs, we obtained a genome completeness of 46.1% with 45–48% of genes annotated. We also found, however, some incongruence in gene annotation of genes/domains recovered in each individual SAG compared with the pooling (Supplementary Fig. S12). Most of the genes/domains that were recovered in one SAG appear as well in the pooling; however there is a fraction of genes/domains (9–14%/2–4%) which remain exclusive to an individual SAG, while another fraction is exclusive to the pooling (10%/7%). This is likely due to the annotation process. It is clear that the three assemblies combined contain the same genomic information than the pooling (see results), however the splits among contigs can be different.

Whether co-assembling different SAGs is a valid method or not remains unclear, since the different SAGs may represent different taxa or strains. Mangot *et al*.^28^ proposed that two genomes with >95% average nucleotide identity (ANI) are similar enough to perform a co-assembly. In our case, the three SAGs present a high pairwise identity among them of 99% (Supplementary Fig. S11), despite having been sampled in different locations (Supplementary Table S4 and Supplementary Table S1). However, each SAG has ~95% of identity with the reference genome of *M*. *brevicollis* (strain MX1) (Supplementary Fig. S3). Therefore, our SAGs can be pooled together, but they likely belong to a different strain of *M*. *brevicollis* than the one previously sequenced^31^. A whole-genome alignment between the pooling assembly and the reference genome revealed few genomic rearrangements between our SAGs and the MX1 strain (Supplementary Fig. S4), affecting 55 scaffolds of the pooling (~800 kb in length) and 48 genes. Although we cannot determine if such inversions are natural or caused by assembly errors, we can nevertheless infer that inversions are relatively infrequent between strains, affecting only 0,71% of the genes found at the pooling assembly. Therefore, taking this into account and the fact that the scaffolds from our SAG assemblies fully align with the reference genome (96.6–100%, Fig. 3), it is clear that the low contiguity of SAG assemblies (N50 of MB1 is 7,754), with fragmented genes (e.g. MB1 has 4,155 genes with only 20–50% of completeness), is caused by the low levels of genome recovery of the SAGs (6–32% in assembled length and 3–39% of CEGMA completeness values). This is consistent with the sparse alignments between SAG and *M*. *brevicollis* scaffolds, which identified multiple blocks of missing data within the genome (Fig. 3a; Supplementary Fig. 4a).

In summary, co-assembly of SAGs is potentially a powerful strategy to recover full genomic information from a given taxa and reduce the blocks of missing data. However, this strategy may not be as straightforward as desired. For example, it is often the case that organisms occupying key phylogenetic positions are not such abundant in the environment^43^, making difficult to get enough cells or SAGs from the same taxa, or even clade. Another option to increase the output of single-cell genomes is the use of metagenomics data^44^. However, again, if the organism is not abundant in the environment, it is going to be unlikely to have enough metagenomics data to improve the assembly. Thus, prior information of a given sample is important to evaluate if it is worth generating SAGs.

Another crucial aspect in single-cell technologies is the potential appearance of contaminants^16^. We found that our SAGs present a low level of contamination, except for one SAG (MB4). In our case, and in contrast to a previous SCG study^27^, the contamination only comes from bacterial origin. This is understandable, given that environmental SAG samples can include ingested, attached, or symbiont bacteria, especially in heterotrophic eukaryotes like choanoflagellates. In addition, cell sorting, cell lysis and WGA must be performed following strict conditions to avoid DNA contamination from other sources^21^. Unexpectedly, our analyses show that our SAGs contain bacteria species not related to marine environments, but rather to freshwater environments or to symbiotic/parasitic bacteria of eukaryotic organisms (Supplementary Table S3). Thus, most likely, our contaminants arised during the WGA step, rather than be truly marine bacteria engulfed by *M*. *brevicollis* cells. It is especially suspicious the presence of bacteria with a 100% identity against *Methylobacterium oryzae* (Supplementary Table S3). In any case, it is important to know which scaffolds from a given assembly come from bacterial origin in order to do not interfere in final results and lead to potential miss-interpretations^45^.

Even though our SAGs recover a few phylogenomic markers from our previous dataset^38^ (7 out of 78 in the worst case, SAG MB2), our phylogenetic analysis placed the SAGs in the expected phylogenetic position^46,47^, that is as sister to *Salpingoeca rosetta* and *Salpingoeca infusionum*, with high statistical support (Fig. 4). Thus, SAGs can be used to better place uncultured protists in the eukaryotic tree of life with the help of a phylogenomic matrix, obtaining better phylogenetic resolution, specially for deep eukaryotic relationships^48^, than the classical phylogenies based on the 18S ribosomal gene. However, phylogenetic questions are not always simple. The use of inappropriate phylogenomic datasets, or the lack of proper taxon sampling can impede to obtain good phylogenies^7^. For example, the incongruences found in the internal phylogeny of choanoflagellates in our trees, which in many cases did not recover the consensus topology^46,47^, can be explained by the lack of enough taxon sampling available, which can lead to low statistical support in the internal nodes. In any case, the broad topology expected^38^ was recovered (Fig. 4), Moreover, and in contrast to 18S ribosomal phylogenies^49^, choanoflagellates monophyly appear highly-supported in our tree (Fig. 4), while filastereans remain as monophyletic clade as previously reported in other studies^38,50^.

Regarding gene annotation, we found that evidence-based gene finders like Augustus perform better than *ab initio* gene predictors. To circumvent the lack of transcriptomic data, Augustus can be trained either with CEGMA or with complete proteins predicted by Snap. Both methods perform similarly. The Jaccard statistic values when comparing the SAGs annotation over the genes of *M*. *brevicollis* is far from an optimal situation (~0.5, meaning 50% overlap). This explains the overestimation of annotated genes: our MB1 annotation contains between 5,610–7,800 proteins, however these proteins correspond only to 3,079–3,110 genes of the reference *M*. *brevicollis* genome. This is due to genes that are split into many genes in SAG annotations that actually belong to the same coding region in the reference genome. Additionally, there are mis-annotations that do not correspond to any gene from the reference genome. Therefore, an annotation strategy aimed at maximizing the number of annotated genes alone can be misleading. Finally, the number of protein domains across SAGs and across different annotation strategies, confirms gene mis-annotations, specially for the *ab initio* annotation performed with Snap. In addition, the total number of different protein domains recovered goes up to 55% in the best case (MB1 Augustus annotation trained with SNAP), higher than the percentage of total genes obtained, which in this annotation was 33% (Fig. 5). Thus, protein domain-based analyses of genome content could be, in principle, more precise than gene annotations.

What can then be done with the data generated from SAGs? Unfortunately with the levels of genome completeness obtained (6–36%), and with genes often appearing fragmented, it is difficult to perform comparative genomics studies of gene gain/loses, gene architectures, or macro- synteny processes with a single SAG alone. However, the recovery of protein domain moves between 30–50% even in SAGs with moderate genomic completeness (15–36%). Therefore, SAGs can reveal an important fraction of the protein domains present in that taxa, which may redefine the evolutionary history of certain gene families. This is especially relevant for uncultured organisms occupying key phylogenetic positions. Furthermore, a phylogenomic analysis of our SAGs have proven to reproduce *M*. *brevicollis* phylogenetic position^38^, even when genome completeness was very low. This indicates that SAGs can be phylogenetically informative, and useful to place uncultured eukaryotic organisms within the tree of life, while potentially revealing new evolutionary insights by the analyses of protein domains.

Finally, there is the question on whether single-cell transcriptomics (SCT) might be a better approach to address evolutionary questions than SCG. Although this remains unsettled, it is true that current data seems to support so. SCT based in Smart-seq. 2^51^ for mRNA retrotranscription and amplification, has shown to recover a third part of the genes on mouse embryonic stem cells^52^. Thus, this represents a similar percentage of gene recovery than the one here obtained in our best SAG, with the advantage that SCT avoids gene annotation problems, given that the full transcript sequence is obtained. In fact, SCT data have been recently used to study the deep evolution of Amoebae^53^. However, it is worth mentioning that mouse or amoebae cells may contain much more RNA than pico-nano sized protist (which are smaller than 20 μm). Therefore, the story with small protists may vary significantly. Additionally, SCT data only allows to obtain the genes that are being expressed through mRNA, hence, the ribosomal genes or other potentially interesting genes that might reveal ancestral functions, but that are not commonly expressed, are not going to be recovered. All in all, both techniques have some drawbacks, and we need more data to properly compare both approaches. An ideal scenario would be the combination of different SAGs (>10), combined with SCT data to assist the genome annotation process and complement the SAGs gene recovery.

## Conclusions

Even though single-cell genomics appeared as a very promising technology, the first analyses with eukaryotes, especially those coming from environmental samples, seemed to put into question its potential given the low genomic recovery. Our data reveals the problems and challenges of working with SCG data and provides a general framework to face them. We believe there are three important issues worth considering, which are selecting promising samples, maximizing the length of final assemblies, and using the best annotation strategies. Our data show that each individual SAG has different biases and genome recovery values, and that genes are often fragmented and even split during the annotation process. In the most optimistic scenario, the genome completeness and gene content recovery moves between 30–40%. However, we here confirm that co-assembling different SAGs coming from the same taxa is a good procedure to increase genome completeness. In addition, and besides the current limitations, we believe there is potential to get important insights from uncultured eukaryotic taxa with only a single SAG. For example, data obtained from SAGs provided enough information to perform phylogenomics studies and, thus, they have the potential to improve the tree of life with the incorporation of uncultured taxa that could not otherwise be included in multigene phylogenetic analyses. Moreover, the analysis of protein domain content was more robust than whole-gene annotations. Single domains are often enough to reconstruct the evolutionary history of particular gene families. Thus, even though new strategies and sequencing chemistries are needed to overcome the known limitations, we believe that SAGs can still provide interesting insights onto evolutionary questions.

## Methods

### Cell collection and whole genome amplification

Cells for single-cell genomics were collected from the surface of the Indian Ocean during the Tara Oceans expedition^54^ and cryopreserved as described before^55^. Flow cytometry cell sorting, single cell lysis and whole genome amplification by Multiple Displacement Amplification (MDA)^17^ were performed at Bigelow Single-cell genomics facility (Boothbay, Maine US), as previously described^9,28,56^ (Table S1). The SAGs obtained were screened by PCR using universal eukaryotic 18S 350 rDNA primers^28^. Those SAGs that had a BLAST identity of >99.5% against the 18S rRNA gene of *Monosiga brevicollis* (alignment length >1Kb) and from three different samples were selected (Supplementary Table S4). A total of 4 SAGs were related with the choanoflagellate *M*. *brevicollis*. Due to DNA quality reasons only three of the four samples were sent to library preparation and sequencing (Supplementary Table S1 and S4). Associated environmental data is summarized in Supplementary Table 1 and more details can be found in PANGAEA^57,58^.

### Library preparation and genome sequencing

Three SAGs (MB1, MB2, MB4) were used for the library construction with Nextera DNA Library Preparation Kit (Illumina). Simultaneous fragmentation and adaptor sequence ligation were performed according to the manufacturer protocol. Briefly, 15 ng input DNA was used as the starting material. The DNA was tagmentated, targeting the insert size of 500 bp and purified using Zymo DNA Clean & Concentrator kit (Zymo Research). Five cycles of PCR were carried out to enrich and perform dual indexing on the tagmented DNA. The final indexed libraries were purified using Agencourt AMPure XP beads (Beckman Coulter). Library validation and quantification were performed on an Agilent 2100 Bioanalyzer with the DNA 1000 assay (Agilent Technologies).

Each library was sequenced using one lane of MiSeq reagent kit v2 (Illumina). The sequencing run was performed according to standard Illumina operation procedures in Paired-end mode, with a read length of 2 × 251 bp and the yield of >11 Gb. Primary data analysis, the image analysis, base calling and quality scoring of the run, was processed using the manufacturer’s software Real Time Analysis (RTA 1.18.54) and followed by generation of FASTQ sequence files by CASAVA.

### Assembly and read mapping to the reference genome

Raw reads obtained were trimmed with Trimmomatic v3.0^59^ using the following options: ILLUMINACLIP:/adapters/NexteraPE-PE.fa:2:40:15 HEADCROP:10 CROP:240 SLIDINGWINDOW:4:28 MINLEN:50. A range between 14–20 million reads were obtained from each SAG, representing a sequencing depth of 70–94X (Supplementary Table S2). Next, these reads were mapped to the reference genome, downloaded from Ensembl Protist V1.0 (http://protists.ensembl.org/Monosiga_brevicollis_mx1/Info/Index) with bowtie2^60^. Read alignment coordinates were converted to position-specific genomic coverage in the reference of *M*. *brevicollis* genome, using SAMtools v.0.1.19^61^ and BEDtools v.2.17.0^62^. R base v3.3.1^63^ was used to plot the coverage of each SAGs reads in a specific region of *M*. *brevicollis* genome. Downsampling calculations were made by extracting randomly a 10%, 30%, 50% and 80% of the reads from each SAGs using seqtk script (https://github.com/lh3/seqtk). Next, a genome assembly for the different amounts of reads extracted was performed with SPAdes^36^ v3.6.1 with the options–sc–careful and -k 21,33,55,77,99. The final assemblies were performed using the same SPAdes options, but with more reads from a less strict trimming: ILLUMINACLIP:/adapters/NexteraPE-PE.fa:2:40:15 HEADCROP:10 CROP:240 SLIDINGWINDOW:6:20 MINLEN:50. Genome statistics were obtained with QUAST^64^. The percentage of core eukaryotic conserved proteins was calculated with CEGMA^29^.

### Scaffold mapping to the reference genome

SAGs scaffolds were aligned to the reference genome using LAST^65^, and the alignment coordinates were converted to BED format using SAMtools. For visualization the Integrative genome viewer, IGV^66^, was used. Only the scaffolds from SAGs assemblies bigger than 500 bp were used for the alignment, in order to avoid noisy signal from uninformative short scaffolds and to reduce the computational cost of the analysis. To calculate which percentage of coding regions were recovered in our SAGs, taking into account different lengths of the coding areas recovered, we used the Intersect option from BEDtools, with different thresholds of alignment length (keeping 90%, 50%, and 20% of the gene length).

### Contamination screening

We followed four different procedures to detect and identify possible contaminants in our SAGs. First, we aligned all the non-*M*. *brevicollis* scaffolds against the NCBI nt database using BLASTn (evalue < 10^−5^). These scaffolds were assigned a taxonomy according to the result of the first BLAST hit, and those that did not belong to *M*. *brevicollis* were excluded of the subsequent annotation process. Second, a tetranucleotide-frequency analysis was performed on the scaffolds bigger than 10 Kb and with a window size of 5 Kb thanks to a custom perl script^67^. Frequencies were clustered using ESOM^68^ (Emerging self-organizing maps). Raw data was normalized using robust estimates of mean variance and trained according to a previous study^67^.Third, we confirmed that the GC content distribution did not present two peaks after removing the contaminant scaffolds, and, using the program OcculterCut^69^, that the GC content was similar to the reference genome. Finally, we searched for the presence of prokaryotic 16S rDNA sequences by performing a BLASTn of the 16S rDNA gene sequence on our contaminant scaffolds. We used as a query the sequence of *Legionella parisiensis* (NCBI accession number U59697), as it was the most frequent taxonomy obtained in our contaminant scaffolds. 16S rDNA sequences recovered from each SAG, were joined together and clusterized in OTUs using USEARCH^70^.

### Average nucleotide identity calculations

The average nucleotide identity (ANI) between SAGs and between each SAG with the reference genome of *Monosiga brevicollis* was calculated by BLASTn^71^ with a minimum similarity of 70% and a maximal e-value of 10^−5^ ^28^, in order to only capture homologous regions^72^ and to reduce the number of overlapping alignments that BLAST can produce. These residual overlaped alignments of BLAST ouput were merged thanks to BEDtools^62^, which also calculates the average of identity on these conflictive alignments. Finally, we calculated the weighted average of nucleotide identity taking into account the length of all BLAST alignments. As the alignment lengths can be different between the genome acting as a query, and the genome acting as a database; in the pairwise comparison we calculated the ANI using the coordinates of both genomes, and plotted them in the Supplementary Fig. S3

### Analysis of local genomic rearrangements

In order to detect possible local rearrangements between our SAGs and the *M*. *brevicollis* MX1 strain, we aligned the non-contaminant scaffolds of the pooled assembly (query) against the reference genome (target) using Satsuma v3.1.0a^73^ with default parameters. We then used the SatsumaSynteny module to compute whole-genome synteny blocks, which were manually examined to identify cases of genomic rearrangements. Specifically, we retrieved the list of scaffolds of the pooled assembly that aligned to both the positive and negative strands (i.e., they contained at least one inverted segment) of the reference *M*. *brevicollis* genome, totaling 55 scaffolds. The genomic coordinates of these discordant alignments were manually examined, in order to identify the breaking points of each inversion in the *M*. *brevicollis* reference genome (i.e., the genomic region comprised between the discordantly aligned blocks). Finally, we used BEDtools intersect module^62^ to obtain the list of reference *M*. *brevicollis* genes that overlapped the manually curated inversion breaking points (48 genes in total). We used Circos^74^ to plot the aligned genomic regions between selected scaffolds of the pooled assembly and *M*. *brevicollis*.

### Phylogenetic analysis

For the phylogenomic analyses, we used the dataset of 78 single-copy protein domains (SCPD78) developed in a previous study^38^. For each of our SAGs assemblies, we recovered the gene markers using the ortholog search algorithm developed by Torruella and co-workers^50^ which uses tBLASTn to extract protein domains from nucleotide data. The list of gene markers found in each SAG is summarized in Fig. 3b.

We performed four independent phylogenomic analyses of the SCPD78 dataset, using each individual SAG and the reference *M*. *brevicollis* assembly. For each individual gene marker, we produced an alignment using MAFFT^75^ v7.299b L-INS-I with 1000 iterations. Ambiguously aligned positions were trimmed using trimAl^76^ v14, with the automated 1 algorithm. The trimmed SCPD alignments were concatenated with Geneious^77^ v8.0.5. Final alignment details are explained in the results section. The best substitution model for phylogenetic inference was selected using IQ-TREE^78^, using the TESTNEW model selection procedure and following the BIC criterion. In all four cases, the LG substitution matrix with a 8-categories free-rate distribution^79^ (a modification of the standard Γ distribution) was selected as the best-fitting model. Maximum likelihood inferences were performed with IQ-TREE, and statistical supports were drawn from 1,000 ultrafast bootstrap values with a 0.99 minimum correlation as convergence criterion^80^, and 1,000 replicates of the SHlike approximate likelihood ratio test^81^.

### Gene annotation

Three different strategies were used to annotate our SAGs scaffolds, after removing the potential contamination. First, we used the SNAP *ab initio* predictor (based on hidden Markov models for gene structure), run in three iterations and using the output of each step as a training set for the next one (the first SNAP prediction was done using the standard minimal HMM)^39^. Second, we trained Augustus^40^ using protein and mRNA predictions from the previous SNAP annotation (only full CDS, mapped to the genome with Scipio 1.4, BLAT and GMAP^82–84^), followed by an optimization round of the species-specific parameters. Third, we repeated the Augustus training and prediction based on the proteins from the CEGMA dataset. These annotations were mapped to the reference *M*. *brevicollis* genome using PASA^85^ (minimum of 90% identity and 75% transcript coverage). Overlap with the reference genes of *M*. *brevicollis* was assessed with the Jaccard statistic calculated by BEDtools.

A BLASTp^71^ (evalue < 10^−5^ identity >90%) search was used to detect the presence of reference *M*. *brevicollis* genes in our SAG annotations. Protein domain annotations of each SAG and the reference *M*. *brevicollis* proteome were computed using Pfamscan and the 29th release of the Pfam database^86^.

### Data availability

Raw reads can be found at ENA, project accession number PRJEB19365. Final assemblies, list of scaffolds classification (whether they were identified as *Monosiga brevicollis* ones or not), the 16S rDNA OTU sequences found in our SAGs and the final alignments and trees obtained in the phylogenetic analysis are available at Figshare (https://figshare.com/articles/Supplementary_material_-_L_pez-Escard_et_al_2017/4629670).

## Electronic supplementary material

Supplementary Information
