# Supplementary material for: Evaluation of single-cell genomics to address evolutionary questions using three SAGs of the choanoflagellate Monosiga brevicollis
Source: Sci Rep. 2017 Sep 8;7:11025. doi: 10.1038/s41598-017-11466-9 (PMC5591225; doi:10.1038/s41598-017-11466-9)
Supplement: Supplementary file 1 — Supplementary Information [file 41598_2017_11466_MOESM1_ESM.pdf]

Supplementary information

Evaluation of single-cell genomics to address evolutionary questions using three SAGs of the choanoflagellate *Monosiga brevicollis*

David López-Escardó[1], Xavier Grau-Bové[1,2], Amy Guillaumet-Adkins[3,4], Marta Gut[3,4], Michael E. Sieracki[5] & Iñaki Ruiz-Trillo[1,2,6*]

Author affiliations:

[1]*Institut de Biologia Evolutiva (CSIC-Universitat Pompeu Fabra), Passeig Marítim de la Barceloneta 37-49, 08003 Barcelona, Catalonia, Spain.*

[2]*Departament de Genètica, Microbiologia i Estadística, Universitat de Barcelona, Barcelona, Catalonia, Spain.*

[3]*CNAG-CRG, Centre for Genomic Regulation (CRG), Barcelona Institute of Science and Technology (BIST), Barcelona, Spain.*

[4]*Universitat Pompeu Fabra (UPF), Barcelona, Spain.*

[5]*National Science Foundation; Arlington, VA, USA*

[6] *ICREA, Pg. Lluís Companys 23, 08010 Barcelona.*

* Correspondance and request for material should be addresed to I-RT (inaki.ruiz@ibe.upf-csic.es)

Supplementary Tables

Table S1: Main environmental features of the SAG samples send to sequencing:

SAG	TARA station	Coordinates	Date	Depth (m)	Temp. (ºC)	Oxygen (µmol/kg)	Salinity (psu)	Chlorophyll (mg Chl/m3)
MB1	39	18.5918ºN 66.6220ºE	18-3-2010	5.4	26.8	193.4	36.3	0.1
MB2	46	0.6625º S 73.1610ºE	15-4-2010	5.5	30.1	185.7	35.1	0.12
MB4	41	14.5536º N 70.0128ºE	30-3-2010	58.2	27.1	148.3	36.5	0.48

Table S2: Read information and genome statistics from the assemblies performed in each SAG

SAG	Assembly	Number of reads	Assembly length (Mb)	Coverage†	N50	L75	Largest scaffold (bp)	Number of scaffolds*	LAST mapping (%)Ω
MB1	Downsampling 10%	3.04E+06	7.61	15X	3,214	1,554	83,973	4,173	86.4
	Downsampling 30%	9.13E+06	11.2	44X	4,911	1,561	91,668	4,933	87.3
	Downsampling 50%	1.52E+07	12.94	73X	5,256	1,787	106,725	5,339	88.1
	Downsampling 80%	2.43E+07	14.7	117X	5,796	1,928	153,330	5,706	88.5
	Downsampling 100%	3.04E+07	15.44	146X	5,520	1,807	177,685	5,842	86.2
	**Final Assembly**	**3.55E+07**	**17.14**	**171X**	**7,754**	**1,613**	**212,553**	**5,959**	**87.9**
MB2	Downsampling 10%	2.44E+06	0.98	12X	3,287	194	20,826	526	100.0
	Downsampling 30%	7.33E+06	1.44	35X	4,682	230	20,71	687	100.0
	Downsampling 50%	1.22E+07	1.69	59X	5,016	240	26,431	759	99.3
	Downsampling 80%	1.95E+07	1.96	94X	4,873	286	25,897	888	97.9
	Downsampling 100%	2.44E+07	2.05	117X	4,631	301	25,221	898	95.6
	**Final Assembly**	**2.99E+07**	**2.49**	**143X**	**7,086**	**380**	**27,988**	**1,212**	**97.7**
MB4	Downsampling 10%	3.40E+06	3.15	16X	3,023	661	54,571	1,768	75.7
	Downsampling 30%	1.02E+07	4.79	49X	4,086	766	101,831	2,331	81.2
	Downsampling 50%	1.70E+07	5.59	82X	4,492	804	107,734	2,538	78.9
	Downsampling 80%	2.72E+07	6.5	131X	5,231	835	86,610	2,788	79.9
	Downsampling 100%	3.40E+07	6.98	163X	5,498	844	93,263	2,852	80.9
	**Final Assembly**	**3.87E+07**	**7.78**	**186X**	**4,585**	**850**	**96,989**	**3,062**	**80.8**

†Times that the genome of *Monosiga brevicollis* is covered by the given number of reads
*Scaffolds bigger than 500 base pairs
Ω Percentage of assembly positions (including contaminant scaffolds) that map with the reference genome of *M. brevicollis*

Table S3: Summary of the 16SrDNA OTUs found in our SAGs including sequence information from the first blast hit againts Genbank:

16S rRNA OTUs	SAG	Accession number	Blast Identity (%)	Taxonomoy	Environment
OTU_1	MB1, MB4	JQ800957	93	Uncultured	Freshwater soil
OTU_2	MB1	JQ684177	99	Alpha-Proteobacteria	Parasite/Symbiont
OTU_3	MB1	JX317612	99	Bacteriodetes	Freshwater
OTU_4	MB1, MB2	LC132814	98	Polynucloebacter	Freshwater
OTU_5	MB2	KP949435	90	Uncultured	Parasite/Symbiont
OTU_6	MB2	JX493275	98	Uncultured	Soil
OTU_7	MB2	LC191539	100	Methylobacterium oryzae	Parasite/Symbiont
OTU_8	MB1	AM936357	98	Delta proteobacteria, Desulfuromonadales	Soil

Table S4: Single-amplified genomes source and technical information

SAG	SAG TARA ID	Station	Cp* (h)	Sequencing
MB1	AB196_C23_CHOA_H	39	8.6	Yes
MB2	AB535_D16_CHOA_H	46	6.12	Yes
MB3	AB241_P18_CHOA_H	41	12.18	No
MB4	AB536_M05_CHOA_H	41	14.97	Yes

*The number of hours to reach above the background fluorescence threshold during WGA

Supplementary Figures

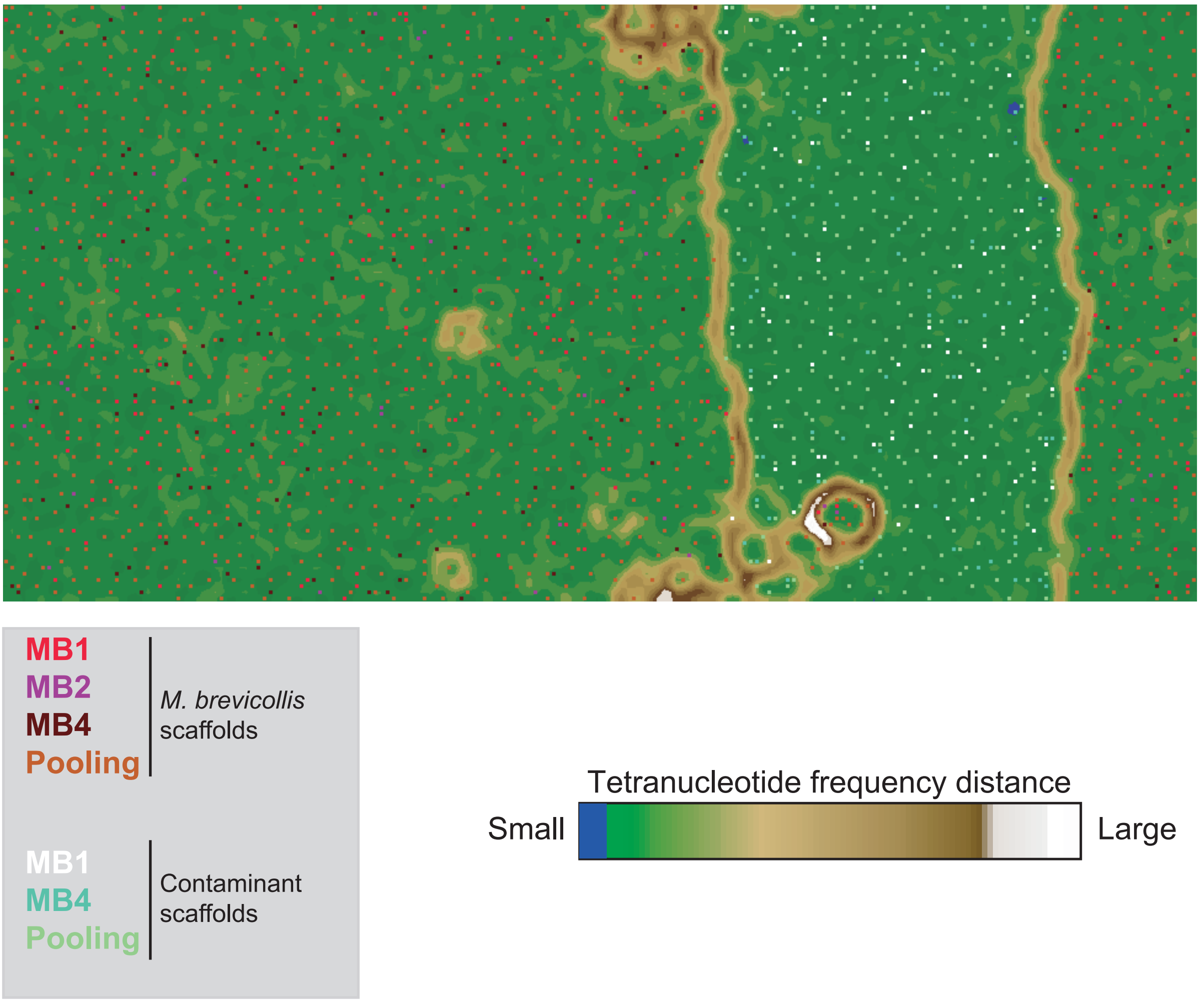

Supplementary Figure S1. Tetranucleotide frequency analyisis represented in an ESOM map. Each scaffold window (<5Kb) is represented by a dot. Dot color marks the scaffold classification. Scaffolds in each assembly were separated corresponding to *M.brevicollis*: MB1 (red), MB2 (purple), MB4 (Garnet) and the Pooling (dark orange); and corresponding to contaminants: MB1 (white), MB4 (light blue), Pooling (light green). MB2 contaminat scaffolds were smaller than 10kb, then excluded for the analysis. Note that dots from different colors have a pattern surrounded by high differences of tetranucleotide frequency (represented in brown/white, scale bar below), where contaminant and *M.brevicollis* scaffolds are separated.

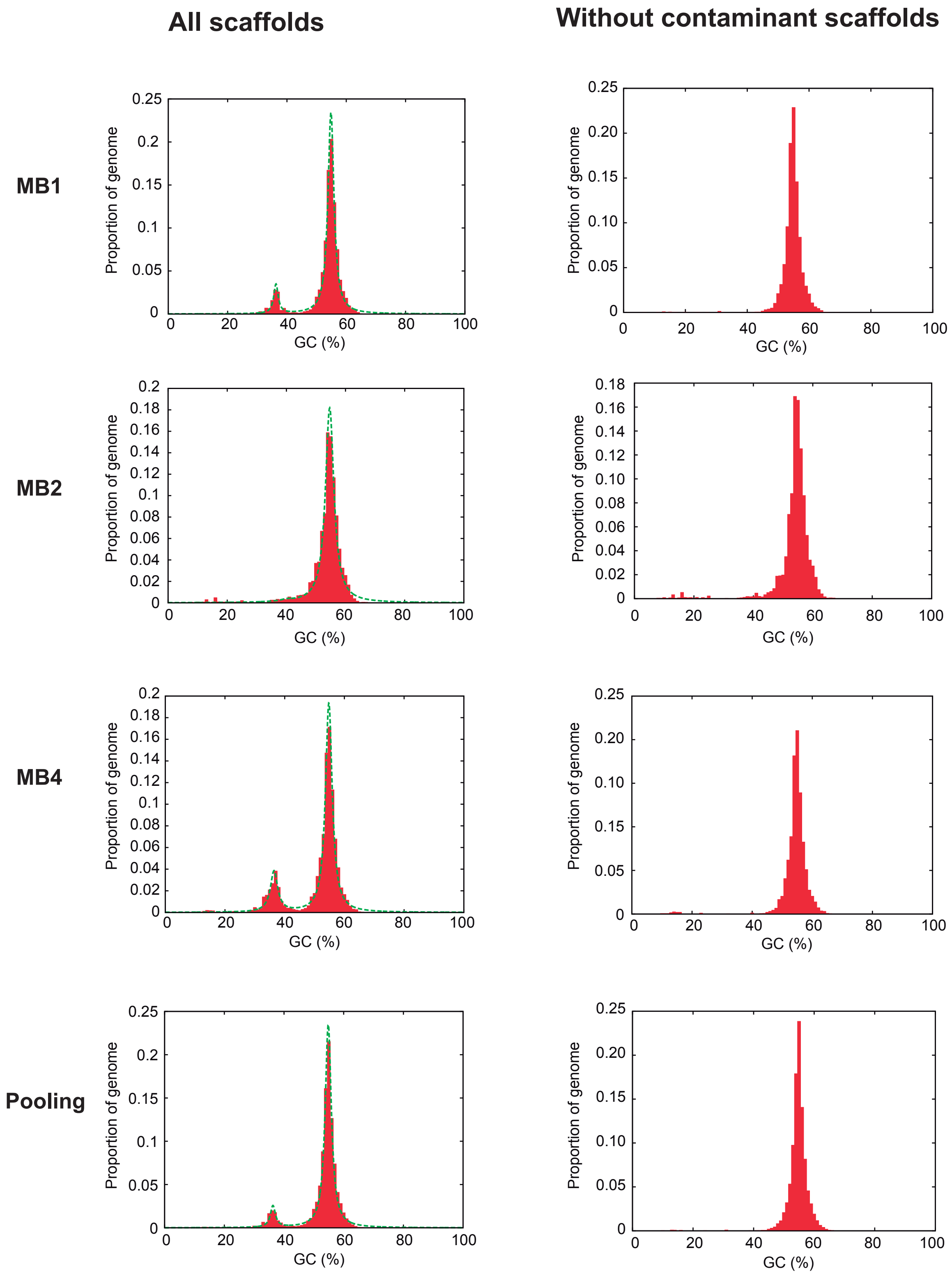

Supplementary Figure S2. GC content distribution. GC content distribution in each final assembly (left) and the distribution after removing contaminant scafolds (right)

Pairwise Identity	MB1	MB2	MB4	M. bre
MB1		99.5	99.1	95.2
MB2	99.5		98.8	94.9
MB4	99.1	98.8		95.2
M. bre	95.1	94.8	95.0	

Supplementary Figure S3. Average nucleotide identites among different SAG assemblies and the genome of *M.brevicollis*. Each average nucleotide identity (ANI) was calculated from both genomic perspectives of the pair (see methods). The column indicates under which coordinates of the given assembly the ANI was calculated. ANI values were obtained through BLASTn alignments, performed with the following thresholds: identity >70% evalue < 10-5.

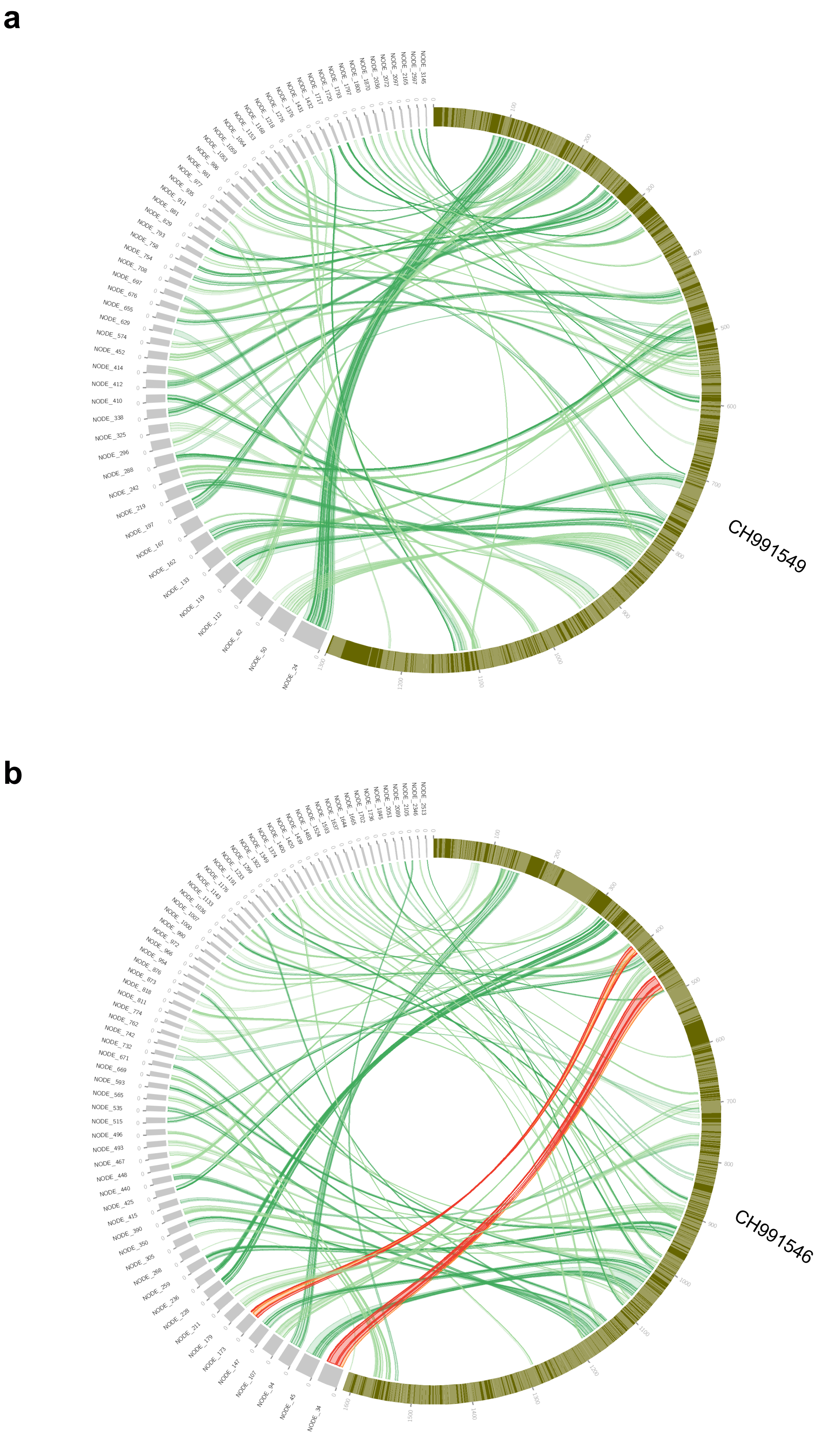

Supplementary Figure S4. Synteny of the pooling assembly and the reference genome of *M.brevicollis*. Both figures a) and b) represent the synteny of the pooling (left) and the *Monosiga brevicollis* (right). Note that *M.brevicollis* scaffolds are depicted with dark and light brownish green, representig the coding regions (light) and the non-coding regions (dark) of the reference genome. Each plot shows one scaffold of the genome of *M. brevicollis,* CH991549 (a) and CH991546 (b). The pooling assembly contains shorter scaffolds that align with the longer scaffold of the reference genome. However they do not cover all the positions from the reference genome. The scaffolds from the pooling that do not present internal inversions compared with *M.brevicollis* genome are depicted in green (in light same direction, in dark the scaffold is oriented backwards). In a) there are not internal inversions events, however in b) there are scaffolds from pooling assembly that present internal inversions, they are depicted in red (backwards orientation) and in orange (same orientation than the reference genome). The length of the scaffolds is expressed in kb.

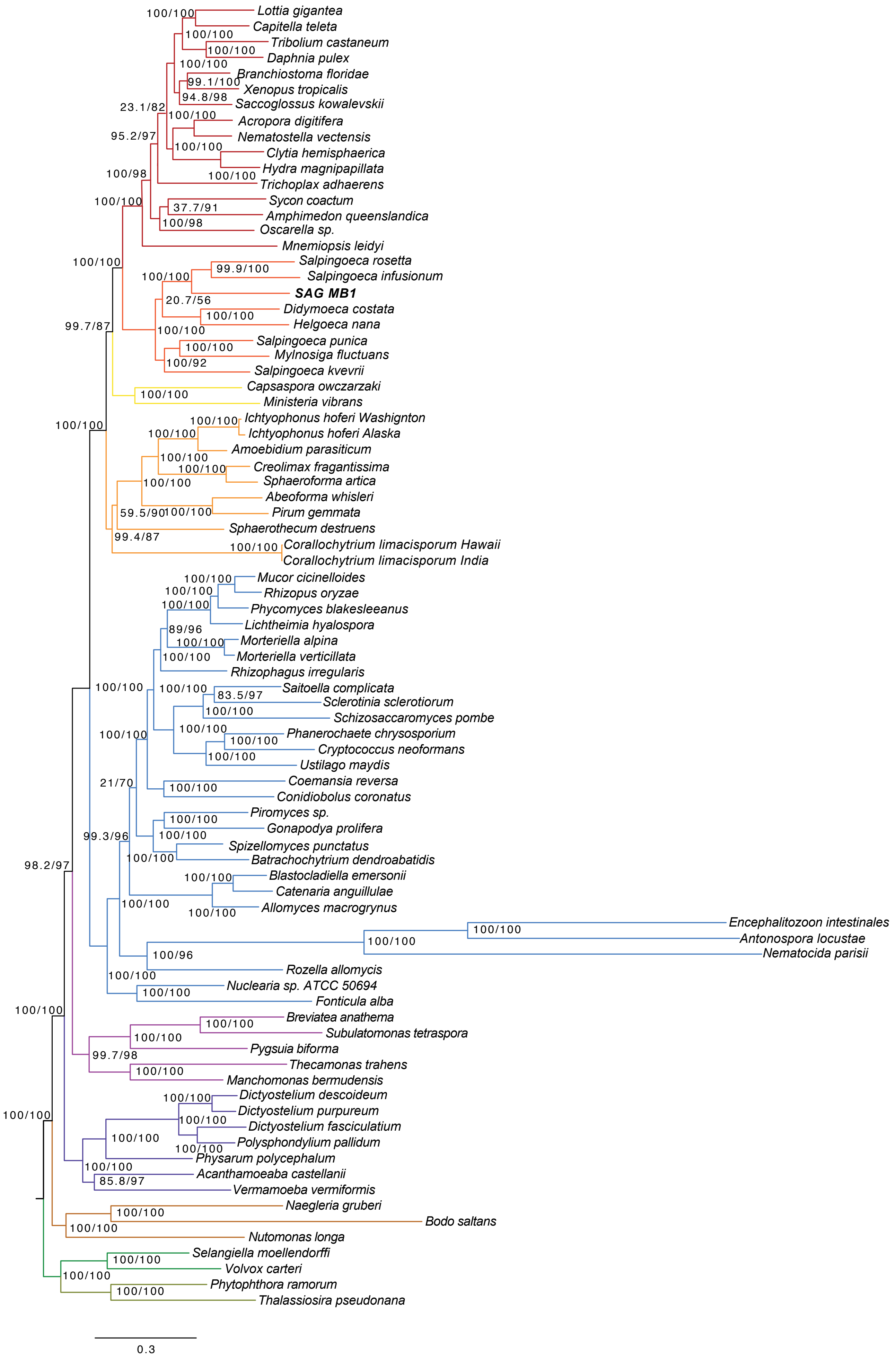

Supplementary Figure S5. Phylogenetic placement of the SAG MB1. Phylogenetic tree based on 82-taxa matrix from our phylogenomic dataset plus the SAG MB1 instead of *M .brevicollis* inferred by Maximum likelihood under the LG+ Γ free rate with 8 categories model. Split supports are bootstraps of single branch test (SH-aLRT, left number) and ultrafast bootstraps (right number) calculated with IQ-TREE.

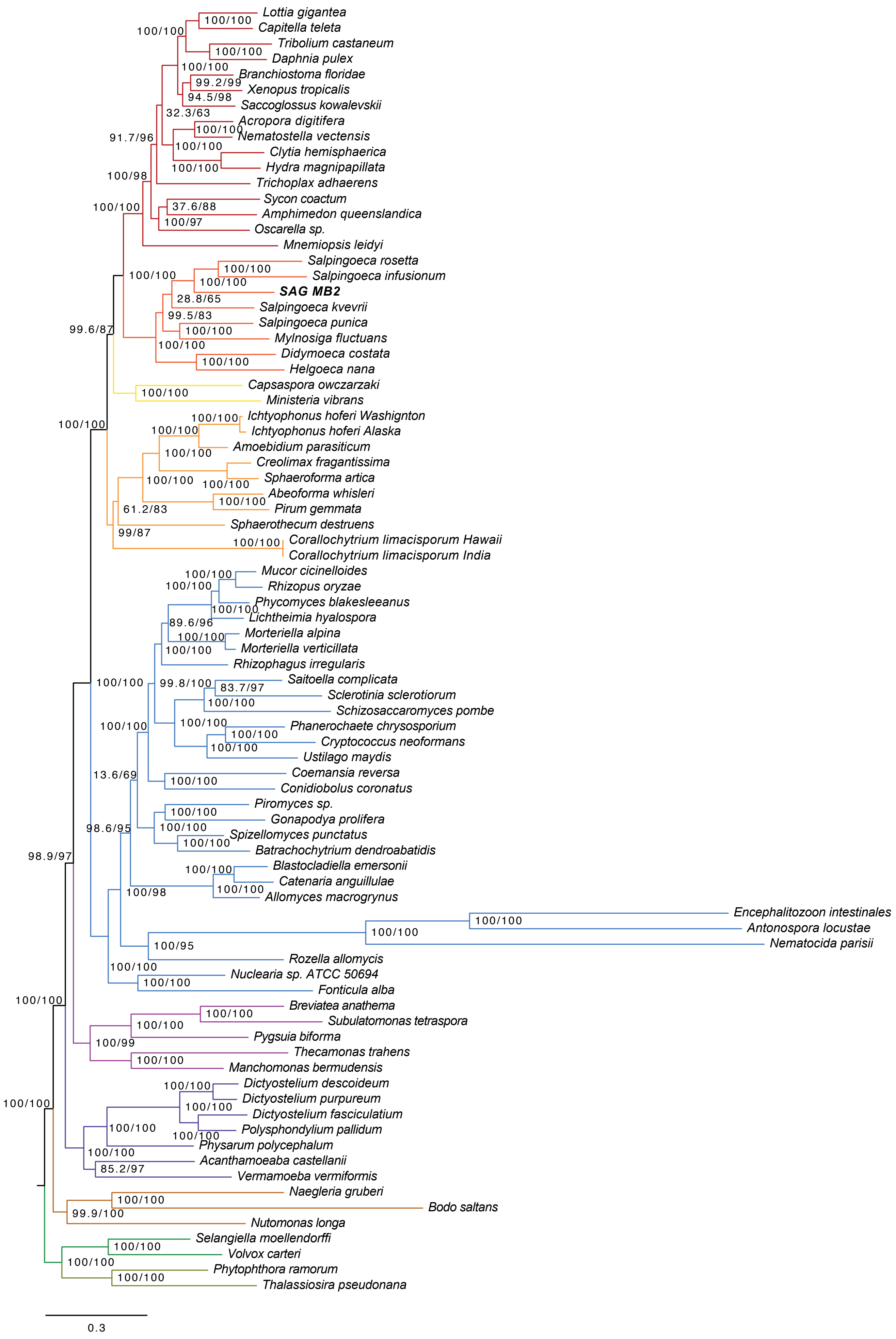

Supplementary Figure S6. Phylogenetic placement of the SAG MB2. Phylogenetic tree based on 82-taxa matrix from our phylogenomic dataset plus the SAG MB2 instead of *M .brevicollis* inferred by Maximum likelihood under the LG+ Γ free rate with 8 categories model. Split supports are bootstraps of single branch test (SH-aLRT, left number) and ultrafast bootstraps (right number) calculated with IQ-TREE.

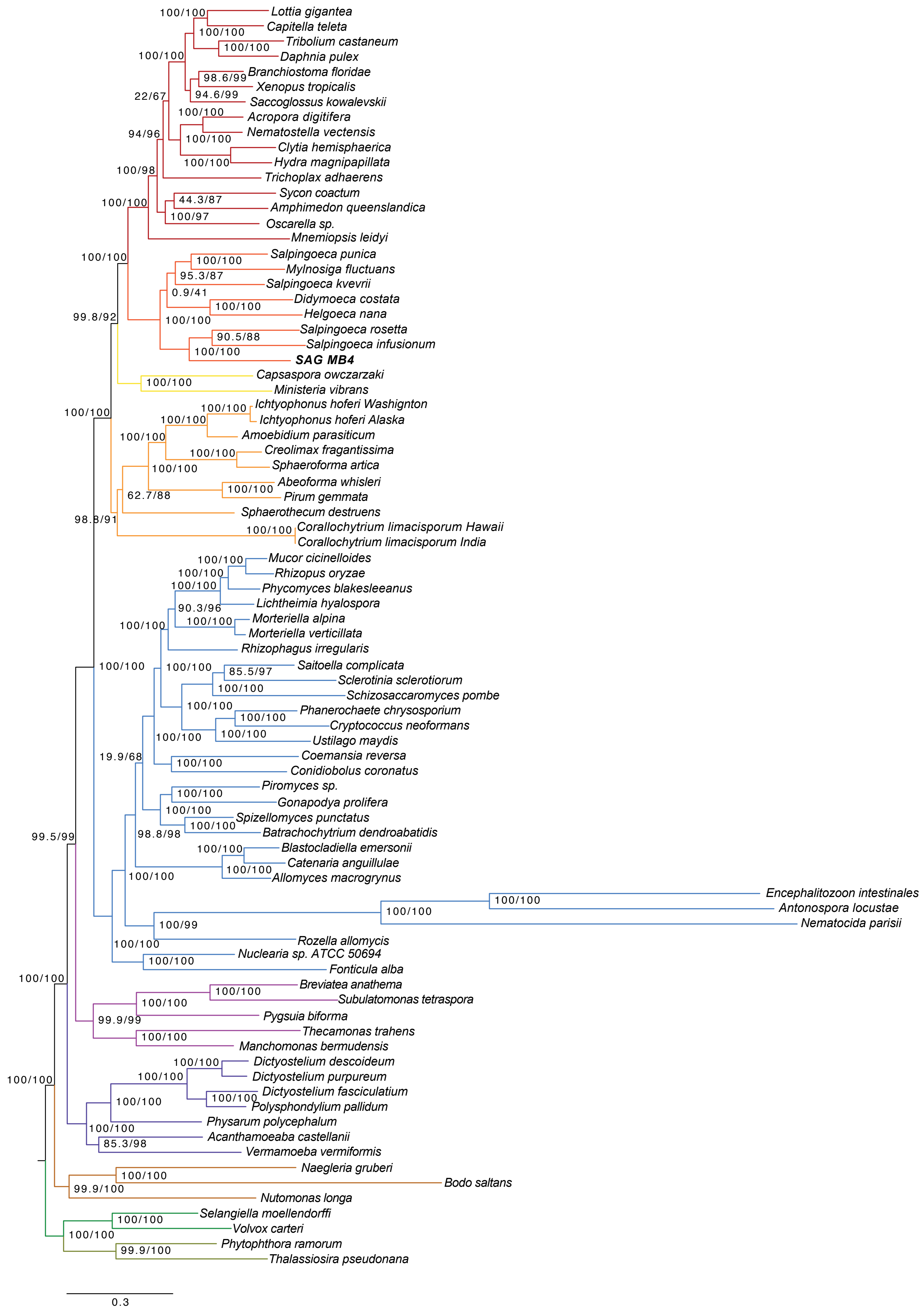

Supplementary Figure S7. Phylogenetic placement of the SAG MB4. Phylogenetic tree based on 82-taxa matrix from our phylogenomic dataset plus the SAG MB4 instead of *M .brevicollis* inferred by Maximum likelihood under the LG+ Γ free rate with 8 categories model. Split supports are bootstraps of single branch test (SH-aLRT, left number) and ultrafast bootstraps (right number) calculated with IQ-TREE.

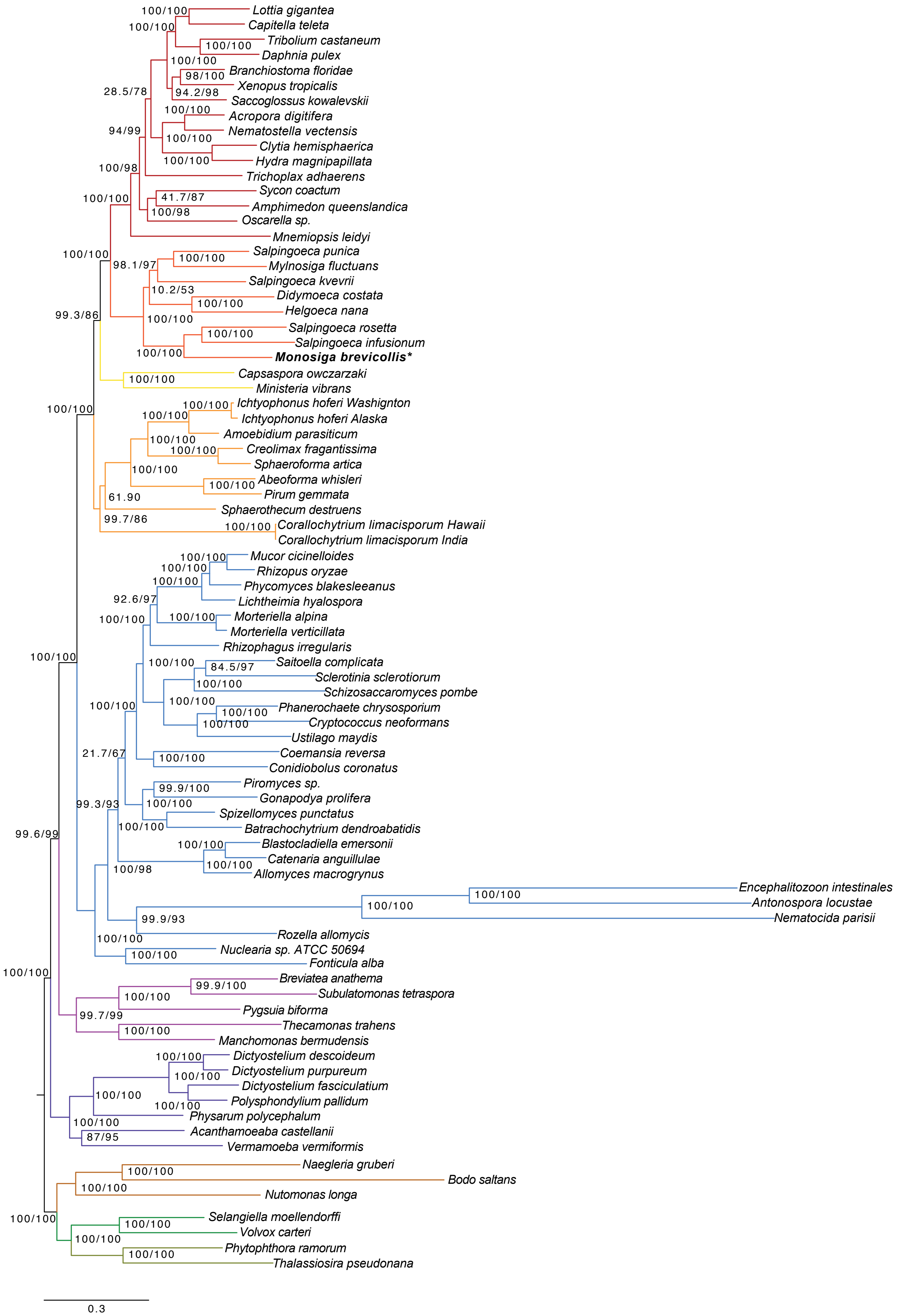

Supplementary Figure S8. Phylogenetic placement of Monosiga brevicollis. Phylogenetic tree based on 83-taxa matrix from our phylogenomic dataset inferred by Maximum likelihood under the LG+ Γ free rate with 8 categories model. Split supports are bootstraps of single branch test (SH-aLRT, left number) and ultrafast bootstraps (right number) calculated with IQ-TREE.

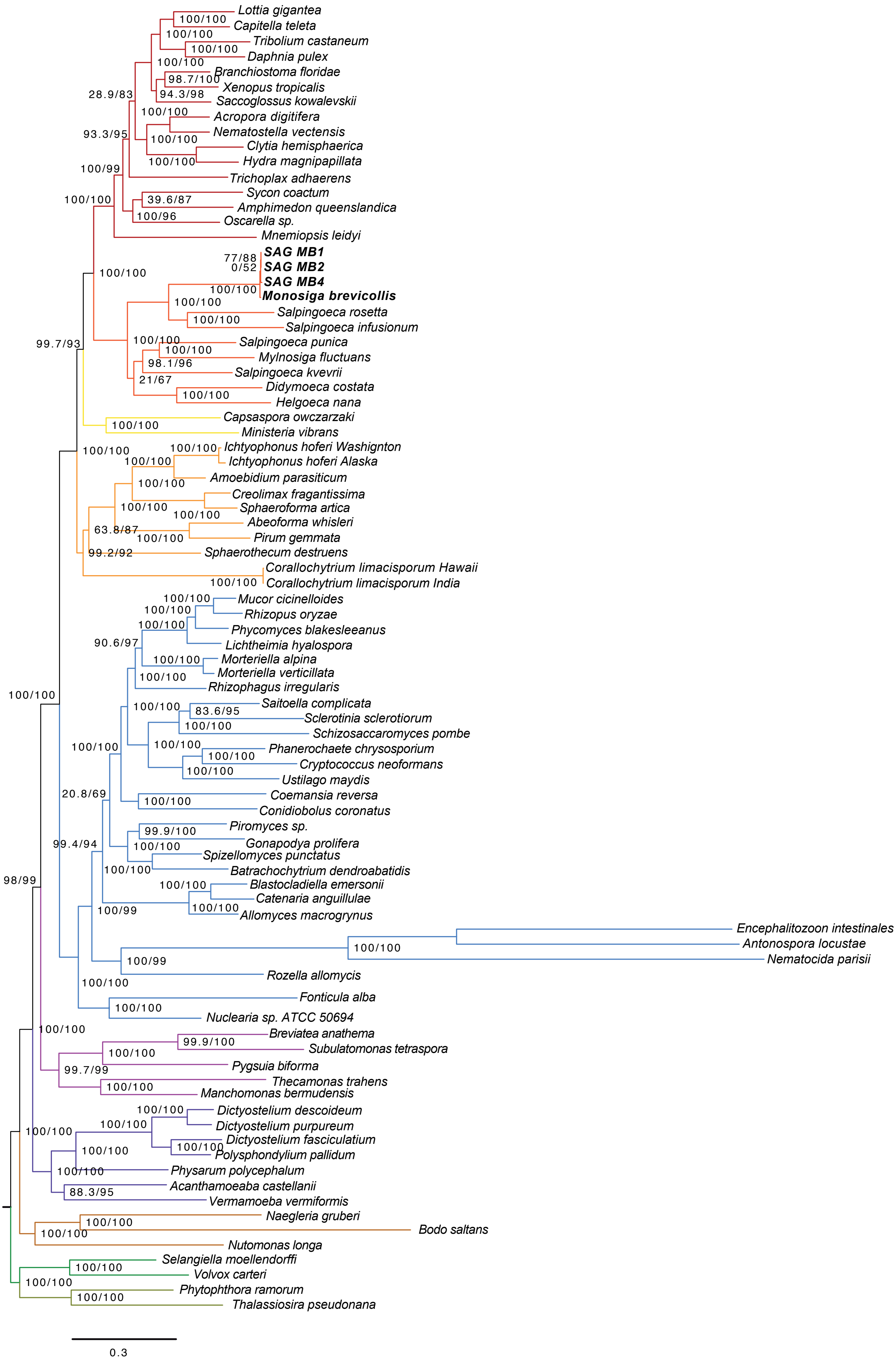

Supplementary Figure S9. Phylogenetic placement of the SAGs and the reference genome of *M. brevicollis*. Phylogenetic tree based on 82-taxa matrix from our phylogenomic dataset plus all the SAGs MB1, MB2, MB4 and the reference genome of *M .brevicollis,* inferred by Maximum likelihood under the LG+ Γ free rate with 8 categories model. Split supports are bootstraps of single branch test (SH-aLRT, left number) and ultrafast bootstraps (right number) calculated with IQ-TREE.

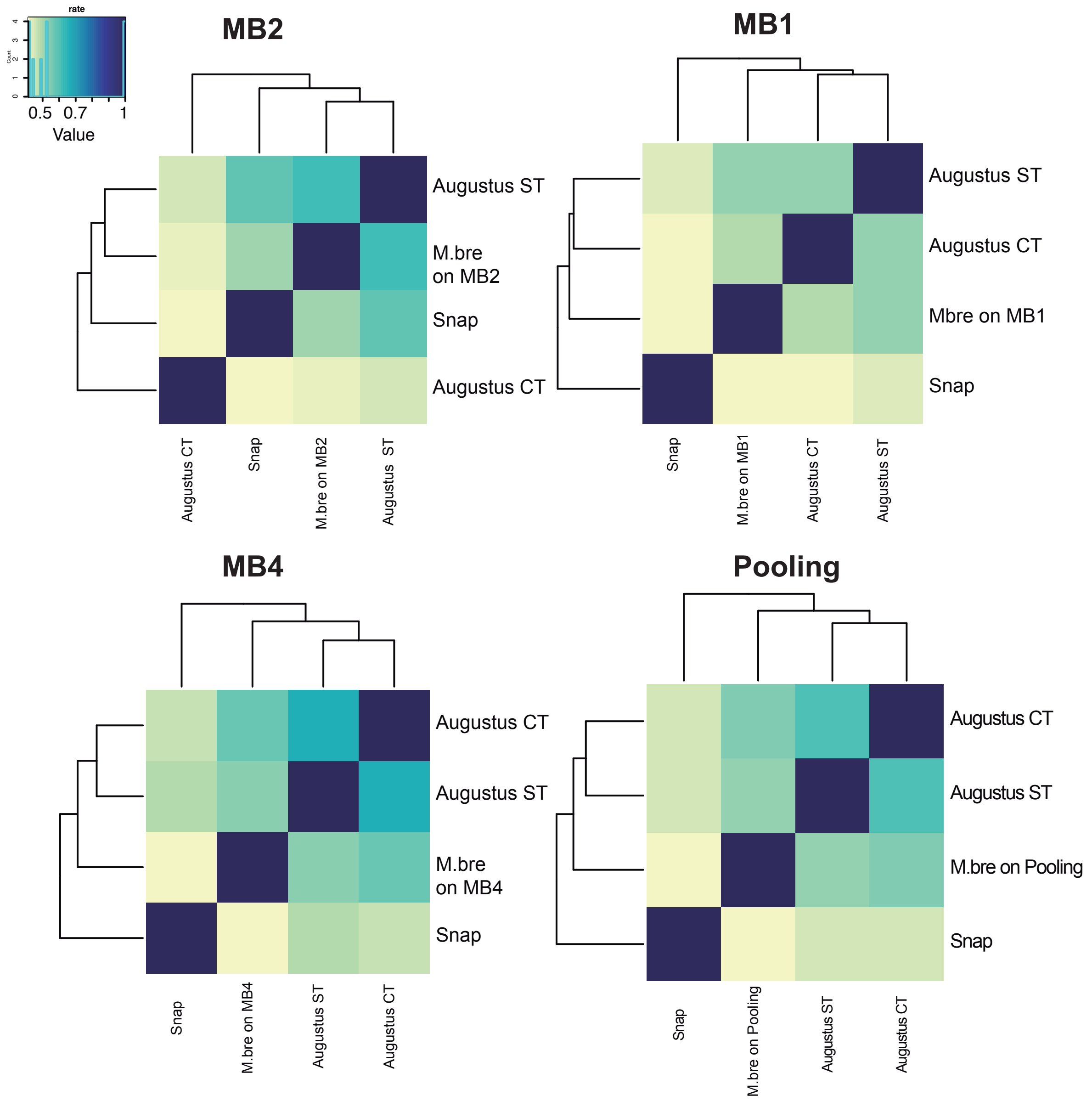

Supplementary Figure S10. Overlap between annotation strategies. Overlap is represented by Jaccard values (0: no overlap; 1: complete overlap) in the color scale (top left). Annotation strategies have been clustered based on pairwise Euclidean distances and a complete linkage clustering algorithm. The reference annotation of *M. brevicollis* over each assembly is also included (labeled “Mbre on ...”). CT: trained with CEGMA proteins. ST: trained with complete SNAP predicted proteins.

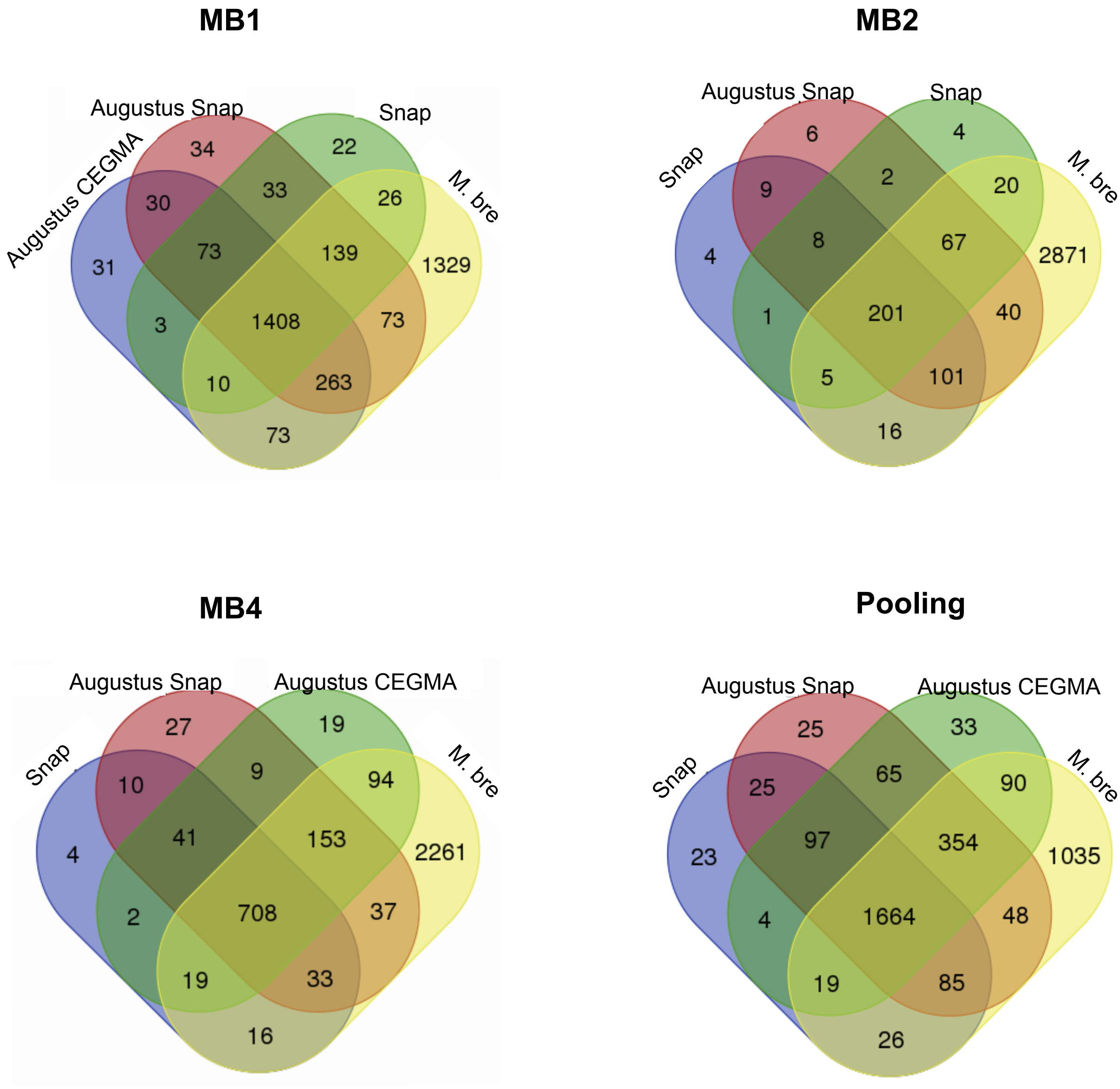

Supplementary Figure S11. Protein domains shared in each annotation. Venn diagram representing shared domains among different annotations strategies on each assembly

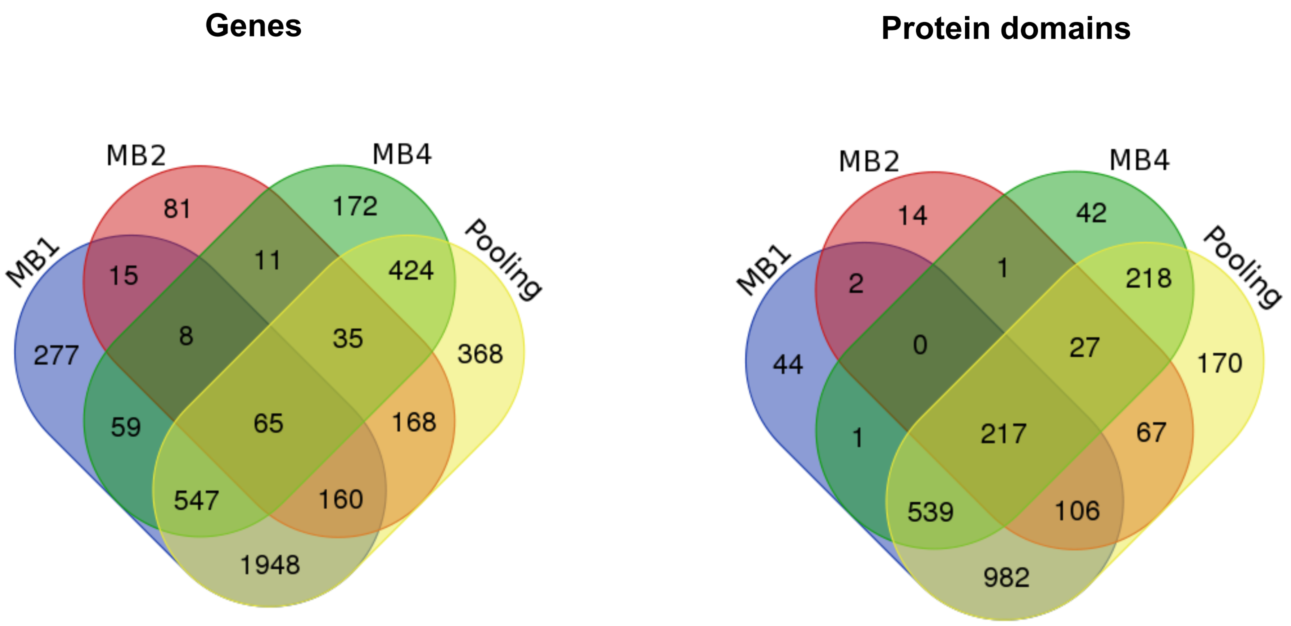

Supplementary Figure S12. Gene and protein domains shared among each assembly. Venn diagrams representing shared predicted genes (left) and protein domains (right) among the different assemblies. Annotation: Augustus trained with complete proteins predicted by SNAP.
